# Predictors of shunt-dependent hydrocephalus following aneurysmal subarachnoid hemorrhage: a pilot study in a single Egyptian institute

**DOI:** 10.1186/s41983-018-0015-1

**Published:** 2018-04-25

**Authors:** Hashem M. Aboul-Ela, Ahmed M. Salah El-Din, Ahmed A. Zaater, Mohamed Shehab, Ossama A. El Shahawy

**Affiliations:** Department of Neurosurgery, Faculty of Medicine, Cairo University, Cairo, Egypt

**Keywords:** Hydrocephalus, Aneurysmal subarachnoid hemorrhage, Bicaudate index, Fisher grade

## Abstract

**Background:**

Acute hydrocephalus can cause neurological deterioration after aneurysmal subarachnoid hemorrhage (aSAH). Predicting which patient would require shunting is challenging.

**Methods:**

This prospective study was conducted upon twenty patients who suffered acute hydrocephalus due to subarachnoid hemorrhage of ruptured aneurysms. Surgical or non-surgical management of hydrocephalus was conducted. Glasgow Coma scale (GCS) was assessed, and hydrocephalus was graded by bicaudate index. Fisher grade was determined from CT scan. Aneurysm site was determined by conventional or CT angiography. Either surgical clipping or endovascular coiling of aneurysms was performed.

**Results:**

Initially, 3 (15%) patients had emergency CSF diversion on admission due to poor GCS on arrival. Initially, the remaining 17 patients were managed conservatively. Five patients did not require any intervention. Twelve patients had external ventricular drainage placement, 4 were weaned, and 8 failed weaning. High bicaudate index (> 0.2) correlated with shunting. Aneurysm site correlated well with shunting (ACoA or PCoA).

**Conclusions:**

Patients with fair GCS can be managed conservatively. Any deterioration warrants shifting to CSF diversion. Higher bicaudate index will usually need CSF diversion. The value of Fisher carries no significant value. Aneurysm location (ACoA or PCoA) correlates with an increased incidence of ventriculoperitoneal shunt placement.

## Background

Acute hydrocephalus can cause early neurological deterioration after aneurysmal subarachnoid hemorrhage (aSAH). Incidence is about 20 to 30%, within 48 h after SAH (Demirgil et al. 2003). Other causes of neurological deterioration after aneurysmal subarachnoid hemorrhage include rebleeding, vasospasm, intraventricular hemorrhage, intracerebral hemorrhage, increased intracranial pressure and edema, seizures, hypoxia, and metabolic disturbances, e.g., hyponatremia. The pathogenesis of hydrocephalus is multifactorial and is related to the obstruction of cerebrospinal fluid (CSF) circulation either within the ventricular system or in the subarachnoid space (Spears et al. 2011). Although pathophysiology of this condition is not fully understood, hydrocephalus after SAH can be treated effectively using current treatment methods. Ventricular drainage may be required either on an emergency basis or can be inserted intraoperatively during aneurysm surgery to achieve adequate brain relaxation. The catheter is converted it to a permanent shunt if it cannot be removed within 5 to 7 days, unless persistent intraventricular blood is present (Klopfenstein et al. 2004). Another alternative often employed is the placement of a continuous lumbar drain in cases of communicating hydrocephalus (Hoekema et al. 2007). Microsurgical fenestration of the lamina terminalis during aneurysm clipping may decrease the incidence of shunt-dependent hydrocephalus (Komotar et al. 2002). This study aims to evaluate the possible predictors for the development of shunt-dependent hydrocephalus in aSAH.

## Methods

This prospective study was conducted upon 20 patients who suffered acute hydrocephalus due to subarachnoid hemorrhage of ruptured aneurysms. Different management modalities, whether non-surgical or surgical, were studied. This study was conducted in the Department of Neurosurgery, Cairo University Hospitals during the period between January 2015 and April 2016.

Patients included in the study were those suffering from SAH due to ruptured aneurysms presenting with acute hydrocephalus or developing in-hospital acute hydrocephalus after admission in the acute event. Patients excluded from the study were those presenting with SAH due to ruptured aneurysms developing hydrocephalus of late onset (more than 1 month after SAH), with no radiological evidence of hydrocephalus during hospital stay. Patients with non-aneurysmal subarachnoid hemorrhage (including perimesencephalic SAH) or with hydrocephalus due to other causes were also excluded.

**Fig. 1:**
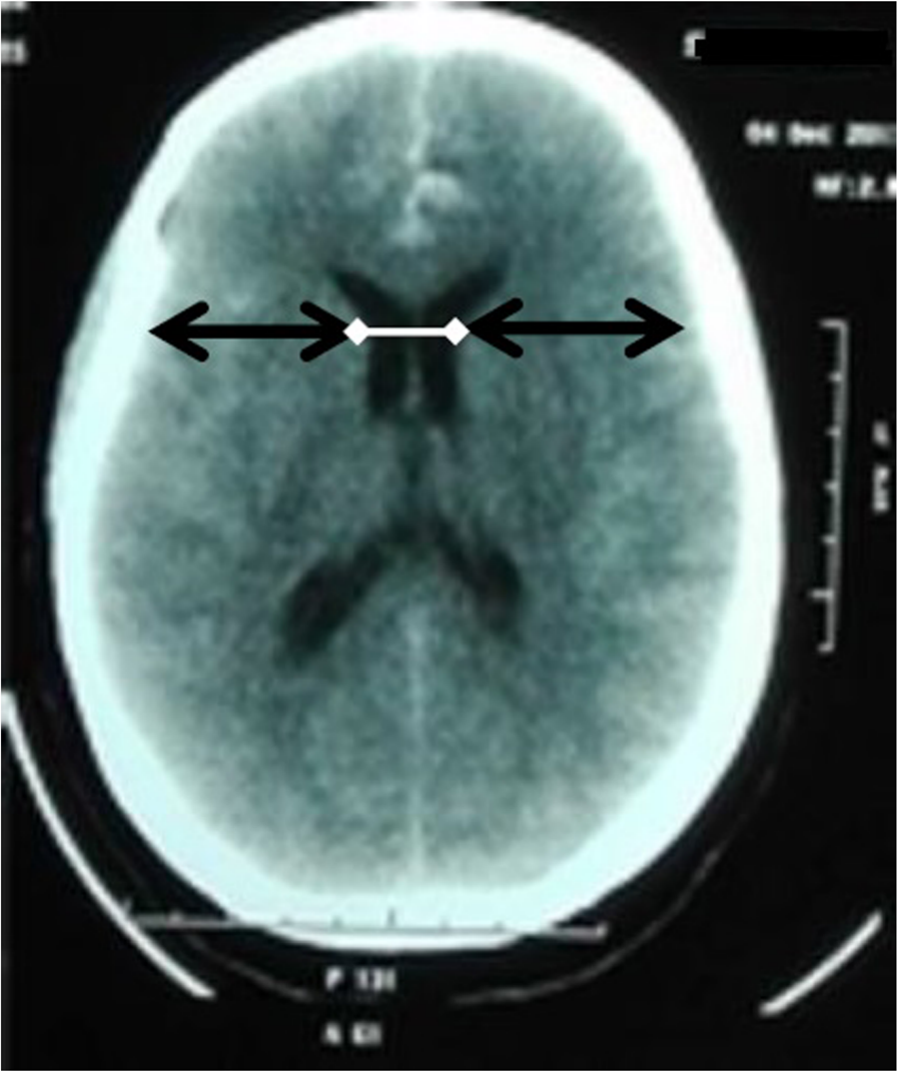
Axial CT scan showing how to determine bicaudate index (white arrow, both black double arrows). White arrow indicates the width of the frontal horns at the level of the caudate nuclei; black double arrows indicate the diameter of the brain at the same level

The patients of the study were subjected to the following:

1. Clinical assessment
  Assessment of level of consciousness according to Glasgow Coma scale (GCS).
2. Investigations
  Computed tomography (CT) scanning for the detection of hydrocephalus and subarachnoid hemorrhage, grading of hydrocephalus by bicaudate index (Fig. 1), and estimation of Fisher grade (Fisher et al. 1980) on CT scan.
  Localization of aneurysm by CT angiography or conventional four-vessel angiography.
  CT scans and angiography were performed using the following machines: General Electric ® Brightspeed (USA) and Siemens ® Somatom Emotion (Germany).
3. Management

Either surgical clipping or endovascular coiling of aneurysms was performed aiming at prevention of rebleeding. In clinically stable patients (GCS 13–15), hydrocephalus was managed conservatively while waiting for the procedure. Close observation of the GCS was carried out in the ICU with serial daily CT scans. Urgent CT scans were performed with deterioration of the level of consciousness (by at least one point) to exclude other causes of disturbed level of consciousness, e.g., rebleeding or expanding hematoma. Deterioration of the level of consciousness warranted shifting to surgical management of hydrocephalus. CSF diversion was performed on an emergency basis in patients deteriorating from acute hydrocephalus by ventriculoperitoneal (VP) shunts, or external drainage or ventriculo-subgaleal shunts whether on admission or during observation period.

### Statistical analysis

Sample size was calculated by GPower 3.1.9.2. (Faul, Erdfelder, Lang, and Buchner—Germany). Using *t* test correlation, point biserial model for effect size of 0.6 with coefficient of determination *p*^2^ for bicaudate index and shunting decision of 0.37 with power of 95% and type I error rate of 0.05 is 20.

Obtained data will be presented as mean or count and percentage as appropriate. Data was computed to measure the association between patient variables and shunting. Analysis of variance according to type of variance data was analyzed using computer package SPSS version 20, 2012 (IBM ®—USA) and Excel version 2013 (Microsoft ®—USA). *P* value ≤ 0.05 will be considered statistically significant. Comparisons were performed using Pearson’s correlation, Fisher’s exact test, and chi-square test for categorical variables and ANOVA for comparison between categorical and continuous variables. Significant variables included in the bivariate analysis (i.e., age, sex, aneurysm location, bicaudate index, Fisher scale, and type of intervention).

## Results

Age of patients ranged from 33 to 76 with mean age of 54.25. Fifty percent of the patients were males and 50% were females. All the patients had anterior circulation aneurysms. The most prevalent type of aneurysm was anterior cerebral artery (ACoA) aneurysm (8 patients—35%) followed by middle cerebral artery (MCA) aneurysm (4 patients—25%) and posterior communicating (PCoA) aneurysm (4 patients—25%); two cases of internal carotid artery (ICA) terminus aneurysm and one patient had multiple aneurysms (bilateral MCA aneurysms and left PCoA). One patient had distal anterior cerebral artery aneurysm (DACA).

Three patients (15%) were treated by endovascular coiling, and the remaining 17 (85%) patients underwent surgery for clipping of the aneurysms. Initially, 3 (15%) patients had emergency VP shunt insertion on admission due to poor GCS on arrival (less than 10 in the 3 patients). The rest of the 20 (85%) patients were managed conservatively while waiting for the definitive procedure for repair of the aneurysms.

Out of the 17 patients, 9 patients (45%) successfully managed without permanent CSF diversion (5 patients did not require any intervention, while 4 patients had external ventricular drainage due to GCS deterioration and were weaned). In the remaining 8 patients (40%), deterioration of GCS lead to external ventricular drainage and failed weaning with subsequent conversion into ventriculoperitoneal (VP) shunts.

### Ventriculoperitoneal shunt predictors

**Fig. 2:**
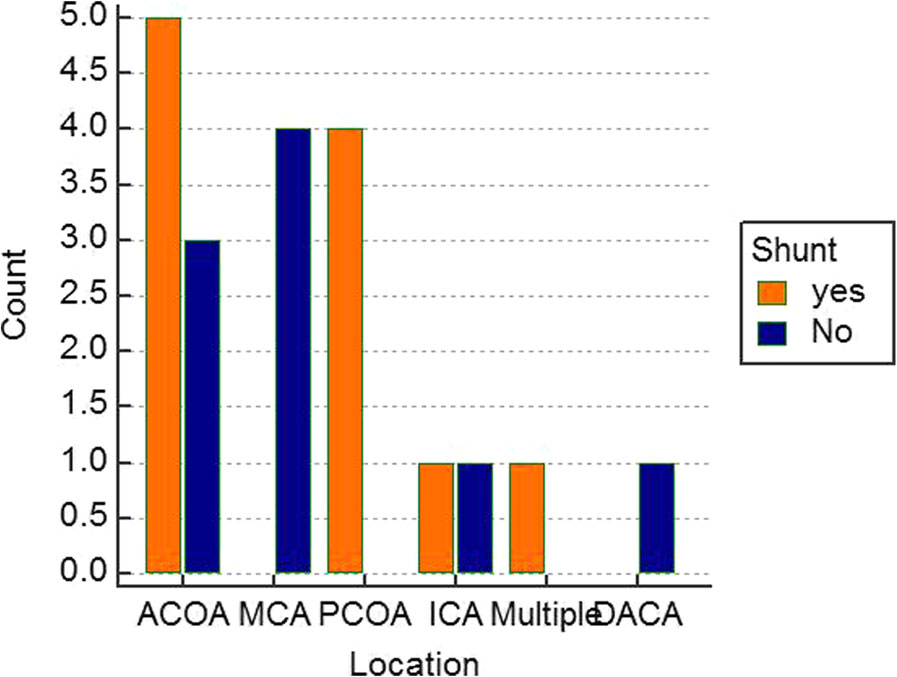
Correlation between shunting decision and type of aneurysm. Abbreviations: CSF cerebrospinal fluid, ACoA anterior communicating artery, MCA middle cerebral artery, PCoA posterior communicating artery, ICA internal carotid artery, DACA distal anterior cerebral artery

The group of patients who underwent shunting procedures was older (mean age was 54.73 compared with 53.67 in non-shunt-treated patients). Shunt dependency was more common in females. Sixty percent of females did not require VP shunt placement, whereas 40% required. Seventy percent of males underwent shunting procedures, while 30% did not. However, correlation of age and sex with shunting decision revealed no statistical significance (*p* = 0.229 and 0.369 respectively) (Table 1). The location of the aneurysm correlated well clinically with the need for shunting with near statistical significance (*p* = 0.0646). 62.5% of patients with ACoA aneurysms (8 patients) required shunting. All the patients with PCoA aneurysms required shunting. All the patients with MCA aneurysms were successfully treated did not require shunting (Fig. 2).

**Table 1:** Predictors for ventriculoperitoneal shunt placement

		No. of shunt-treated patients (total = 11)	Non-shunt-treated patients (total = 9)	Total	*P* value
Mean age		54.73	53.67		0.229
Sex	Males	7 (70%)	3 (30%)	10	
	Females	4 (40%)	6 (60%)	10	
Fisher grade					0.411
2		3 (27.3%)	2 (22.2%)		
3		5 (45.4%)	3 (33.3%)		
4		3 (27.3%)	4 (44.5%)		
Mean bicaudate index at shunt decision	Emergency VP shunt	0.26	0.2		*0.031*
	Failed EVD weaning	0.21			
Aneurysm location	ACoA	5 (62.5%)	3 (37.5%)	24	*0.0646*
	MCA	0	4 (100%)	15	
	PCoA	4 (100%)	0	12	
	ICA	1 (50%)	1 (50%)	6	
	Multiple	1 (100%)	0	3	
	DACA	0	1 (100%)	3	
Type of procedure	Clipping	10 (58.8%)	7 (41.2%)	51	0.566
	Coiling	1 (33.3%)	2 (66.7%)	9	

Italicized *P* values are significant

*Abbreviations*: *CSF* cerebrospinal fluid, *EVD* external ventricular drainage, *ACoA* anterior communicating artery, *MCA* middle cerebral artery, *PCoA* posterior communicating artery, *ICA* internal carotid artery, *DACA* distal anterior cerebral artery

The bicaudate index was measured in the admission CT scan in the 20 patients, and the measurements were as follows: In the group of 8 patients in the shunt-dependent group, the bicaudate index ranged between 0.16 and 0.24 with a mean value of 0.21. In the group of 9 patients who avoided VP shunt placement, the bicaudate index ranged from 0.17 to 0.26 with a mean value of 0.2. In the group of 3 patients who underwent emergent CSF diversion on admission, the bicaudate index ranged from 0.25 to 0.29 with a mean value of 0.26. Therefore, a value above 0.2 correlated with increased shunt dependency. This correlation revealed statistical significance (*p* = 0.031).

The amount of subarachnoid blood was measured from CT scans by using Fisher grade. Fisher grades 3 and 4 in the patients who did not require shunt placement were found in 33.3 and 44.5% of the patients respectively (total 77.8%). Fisher grades 3 and 4 in patients who underwent VP shunt placement were found in 45.4 and 27.3% of the patients respectively (total 72.7%). Accordingly, on correlation, no statistical significance could be found between high Fisher grade and the need for shunting (*p* = 0.411).

## Discussion

### Age and sex

The group of patients who underwent shunting procedures was older but did not reach statistical significance. This correlates well with other studies. In the study by Dorai et al. (2003), the mean age for the study population was 53.2 years (range, 17–89 years). The median age for the shunt-treated population was 60 years, in comparison with 51 years for the non-shunt-treated population. The authors stated a possible explanation; older patients have wider subarachnoid spaces which can hold larger amounts of subarachnoid blood, thus increasing their risk of developing CSF circulation disturbances. Moreover, the ventricular compliance decreases with age leading to the increased liability to symptomatic hydrocephalus (Dorai et al. 2003). The study by Rincon et al. (2010) reported comparable results in which the median age for the shunt-treated group was 59. The median age for non-shunt-treated group was 53 (Rincon et al. 2010).

Gender did not correlate statistically with shunt placement. In the available literature, female sex was more liable for VP shunt placement. In the study by Dorai et al. (2003), 23.8% of the female patients underwent shunting procedures, compared with 15.9% of the male patients. In the study by Chan et al. (2009), 63.5% of the patients who required shunting after failure of external ventricular drainage (EVD) weaning were females and 34.5% were males.

### Aneurysm location

The location of the aneurysm correlated clinically well with the need for shunting. Our results are comparable to the study by Pietilä et al. (1995), in which all the patients with MCA aneurysms did not require VP shunt placement. Nineteen percent of the patients with ACoA aneurysms required shunting, and those came second only to the patients with posterior circulation aneurysms (53%) (Pietilä et al. 1995). Our study did not include posterior circulation aneurysms. In literature posterior circulation, aneurysms are associated with increased incidence of VP shunt placement as in the studies by Dorai et al. (2003), Chan et al. (2009), and de Oliveira et al. (2007). Different aneurysm locations produce different amounts and patterns of bleeding. Posterior circulation and ACoA aneurysms create large amounts of blood in the basal cisterns. The subarachnoid space around them is wide and offers little resistance to extravasation, whereas the narrow sylvian cistern is tighter. Anterior communicating artery and PCoA aneurysms are more often associated with intraventricular hemorrhage (Sethi et al. 2000).

### Bicaudate index

Our results are comparable to the study by Rincon et al. (2010), in which a bicaudate index higher than 0.2 was found to be associated with the need for ventriculoperitoneal shunting. In the study by Little et al. (2008), hydrocephalus was measured by the relative bicaudate index (RBCI) measured on computed tomographic scans at the time of shunting by dividing the bicaudate index by the normal upper age limit. Patients were divided into three groups by ventricle size: group 1 (RBCI less than1.0), group 2 (RBCI between 1.0 and 1.4), and group 3 (RBCI more than 1.4). VP shunt was performed in 16, 49, and 90% of the three groups respectively. Thus, higher bicaudate index correlated more with the need for VP shunt placement. The authors pointed out, however, that the borderline group in the middle ought to be managed more conservatively. These results are comparable to our study (Little et al. 2008).

### Fisher grade

Our results were inconclusive regarding the relationship between high Fisher grade and the need for shunting. Varelas et al. (2006) had comparable results to our study in which they found out that Fischer grade did not correlate with the need for shunting. However, mostly in the available literature, higher Fisher grade correlates with shunt dependency. In the study by Dorai et al. (2003), 19.2% of the patients with thick blood, i.e., Fisher grade 3 required shunting. 10.8% of the patients with thin blood, i.e., Fisher grade 1 required shunting (Dorai et al. 2003). In the study by Dehdashti et al. (2004), in which 245 patients were studied prospectively over 6 years, 33 and 53% of the patients who underwent shunting had Fisher grades 3 and 4 respectively. In the study by de Oliveira et al. (2007), 385 patients were studied retrospectively over a 6-year period and 71 patients (18.4%) required shunting. They found that 93% had Fisher grade 3 (de Oliveira et al. 2007). The study by Gruber et al. (1999) also concluded that high Fisher grade leads to shunt dependency in 26.7 and 32.8% of Fisher grade 3 and Fisher grade 4 respectively.

## Conclusions

Acute hydrocephalus is a serious event complicating aneurysmal subarachnoid hemorrhage. However, thorough monitoring and wise decision making according to the clinical scenario can prevent deterioration and avoid unnecessary CSF diversion.

The most important factor in management remains close monitoring of the level of consciousness. Patients with fair GCS (Little et al. 2008; Varelas et al. 2006; Dehdashti et al. 2004) can be managed conservatively. Any deterioration whether on admission or during expectant management warrants shifting to CSF diversion.

The degree of hydrocephalus as measured by bicaudate index can be beneficial as higher grade will usually need CSF diversion regardless of GCS. The value of Fisher carries no significant value in the management of acute hydrocephalus due to aneurysmal subarachnoid hemorrhage. Aneurysm location (ACoA or PCoA) correlates with an increased incidence of ventriculoperitoneal shunt placement.

Further prospective, randomized trials and experiences will prove useful in determining the most efficacious management algorithm and outline prognostic factors attributed to management of this disease.

## Abbreviations

ACoA: Anterior communicating artery
aSAH: Aneurysmal subarachnoid hemorrhage
CSF: Cerebrospinal fluid
DACA: Distal anterior cerebral artery aneurysm
EVD: External ventricular drainage
GCS: Glasgow Coma scale
ICA: Internal carotid artery
MCA: Middle cerebral artery
PCoA: Posterior communicating
RBCI: Relative bicaudate index
VP: Ventriculoperitoneal
